# Proton pump inhibitor-responsive chronic cough without acid reflux: a case report

**DOI:** 10.1186/1752-1947-1-69

**Published:** 2007-08-25

**Authors:** Kouichi Nobata, Hidetsugu Asanoi

**Affiliations:** 1 The Department of Internal Medicine, Imizu City Hospital, Imizu, JapanAbstract

## Abstract

**Background:**

Because 24-h esophageal pH monitoring is quite invasive, the diagnosis of gastroesophageal reflux disease (GERD)-associated cough has usually been made based merely on the clinical efficacy of treatment with proton pump inhibitor (PPI).

**Case presentation:**

We recently encountered two patients with PPI-responsive chronic non-productive cough for whom switching from bronchodilators and glucocorticosteroids to PPI resulted in improvement of cough. The cough returned nearly to pre-administration level a few weeks after discontinuation of PPI. Though GERD-associated cough was suspected, 24-h esophageal pH monitoring revealed that the cough rarely involved gastric acid reflux. Following re-initiation of PPI, the cough disappeared again.

**Conclusion:**

PPI may improve cough unrelated to gastric acid reflux.

## Background

Gastroesophageal reflux disease (GERD)-associated cough is a well-known type of chronic, non-productive cough [1]. Asthma, postnasal drip syndrome, and GERD are the three most frequently identified causes of cough in Western countries [1]. However, in Japan, GERD-associated cough has been found to account for only a few percent of cases of chronic cough, while cough variant asthma (CVA), atopic cough (AC), and sinobronchial syndrome (SBS) are major causes of chronic cough [2]. Proton pump inhibitors (PPIs) are considered the drugs of choice for acid-related diseases including GERD [3].

**Figure 1:**
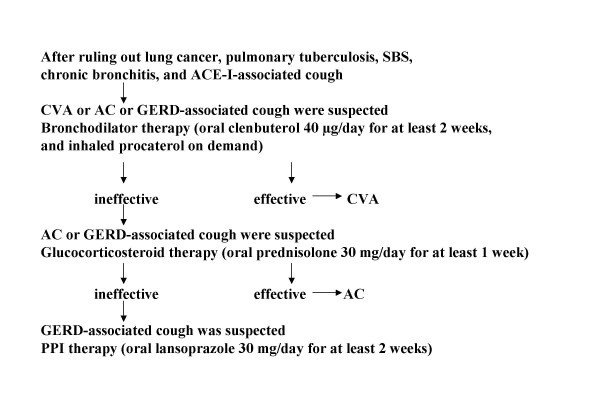
**Assessment of chronic cough lasting more than 8 weeks without history of wheezing**. After ruling out lung cancer, pulmonary tuberculosis, SBS, chronic bronchitis, and ACE-I-associated cough, bronchodilator therapy was initiated (oral clenbuterol 40 μg/day for at least 2 weeks, and inhaled procaterol on demand). If this treatment was effective, CVA was diagnosed. If not, AC or GERD-associated cough was suspected, and glucocorticosteroid therapy was begun (oral prednisolone 30 mg/day for at least 1 week). If this treatment was effective, AC was diagnosed. If not, GERD-associated cough was suspected and PPI therapy was begun (oral lansoprazole 30 mg/day for at least 2 weeks).

In our institutions, chronic cough lasting more than 8 weeks without history of wheezing was assessed as described in Figure 1.

We recently encountered two patients with PPI-responsive chronic non-productive cough in whom 24-h esophageal pH monitoring showed cough rarely associated with a reflux episode. These cases show that PPI may improve cough unrelated to an acid reflux episode.

## Case 1

**Figure 2:**
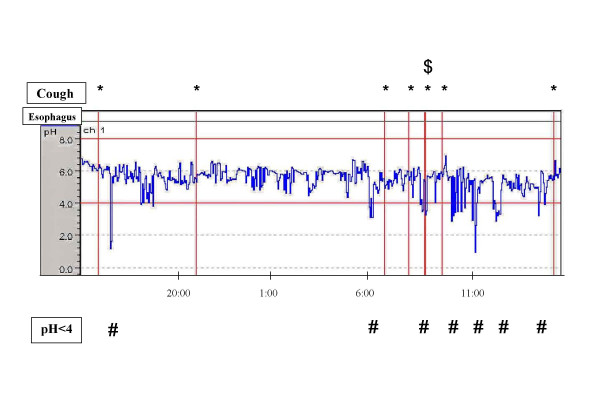
**Results of 24-h esophageal pH monitoring prior to re-initiation of PPI in case 1**. Acid reflux in the esophagus was considered present if pH was 4 or lower. Some cough and acid reflux were observed, little cough-related acid reflux was noted (*; cough, #; acid reflux, $; cough-related acid reflux).

The patient was a 60-year-old man who had been suffering from isolated chronic non-productive cough for about 1 year. He discontinued smoking 9 months before the first visit following development of this cough and had never taken an ACE-I. Although he did not complain of heartburn and other symptoms suggestive of GERD, endoscopic assessment of the esophagus revealed reflux esophagitis (Los Angeles classification Grade B). He had had no respiratory infections during the 8-week period preceding the first visit. No abnormal shadows were noted on chest or paranasal sinus X-rays and chest CT scan. Cutoff points in testing of bronchial hyperresponsiveness and cough reflex hypersensitivity were set at below 10000 μg/ml [4] and 3.9 μM [4]. Airway reversibility to inhaled β2 agonist was 6.5%, and testing for bronchial responsiveness to methacholine and cough reflex sensitivity revealed no hyperresponsiveness (29053 μg/ml) and no hypersensitivity (500 μM). Cell fractionation of bronchoalveolar lavage fluid revealed percentages of macrophages, lymphocytes, neutrophils, and eosinophils of 91%, 7%, 1.7%, and 0.3%, respectively. Cough was evaluated based on frequency and intensity as follows: 10 = cough level at the first visit, 5 = half the level at the first visit, 0 = none. Neither bronchodilator therapy nor anti-inflammatory therapy improved the cough. PPI was given after discontinuing bronchodilator and anti-inflammatory therapy. The cough was markedly improved 2 weeks after initiation of PPI (cough level 1), but returned nearly to pretreatment level 3 weeks after discontinuation of PPI (cough level 7, cough sensitivity 62.5 μM). On 24-h esophageal pH monitoring performed prior to re-initiation of PPI to determine the reason cough improved with PPI, the probe was positioned in the lower esophagus 5 cm above the upper border of the lower esophageal sphincter. Acid reflux in the esophagus was considered present if pH was 4 or less [3]. Some cough and acid reflux were observed, little cough-related acid reflux was noted (Figure 2, *; cough, #; acid reflux, $; cough-related acid reflux). Following re-initiation of PPI, the cough disappeared (cough level 1, cough reflex sensitivity 62.5 μM).

## Case 2

**Figure 3:**
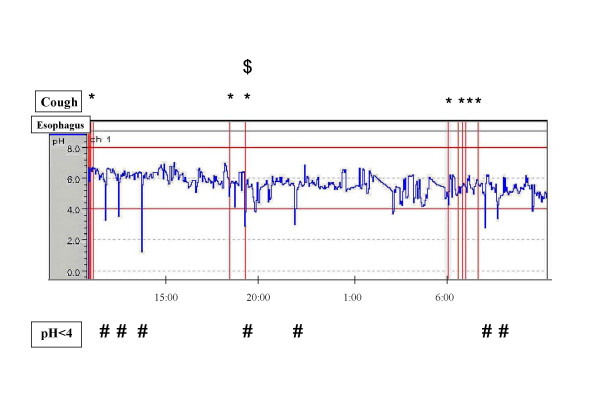
**Results of 24-h esophageal pH monitoring prior to re-initiation of PPI in case 2**. Acid reflux in the esophagus was considered present if pH was 4 or lower. Some cough and acid reflux were observed, little cough-related acid reflux was noted (*; cough, #; acid reflux, $; cough-related acid reflux).

The patient was a 34-year-old man who had been suffering from isolated chronic non-productive cough since 31 years of age. He had stopped smoking at 32 years of age and had never taken an ACE-I. Although he did not complain of heartburn or other symptoms suggestive of GERD, endoscopic assessment of the esophagus revealed reflux esophagitis (Los Angeles classification Grade M). He had had no respiratory infections within the 8-week period preceding the first visit. No abnormal shadows were noted on chest or paranasal sinus X-rays and chest CT scan. Airway reversibility to inhaled β2 agonist was 13%, and testing of bronchial responsiveness to methacholine and cough reflex sensitivity revealed hyperresponsiveness (1208 μg/ml) without hypersensitivity (31.2 μM). Cell fractionation of bronchoalveolar lavage fluid revealed percentages of macrophages, lymphocytes, neutrophils, and eosinophils of 92%, 5%, 3%, and 0%, respectively. Neither bronchodilator therapy nor anti-inflammatory therapy improved the cough. PPI was given after discontinuation of bronchodilator and anti-inflammatory therapy. The cough was markedly improved 1 week after initiation of PPI (cough level 1), but had returned nearly to pre-administration level by 3 weeks after discontinuation of PPI (cough level 8). On 24-h esophageal pH monitoring performed prior to re-initiation of PPI, some cough and acid reflux were observed, but little cough-related acid reflux was noted (Figure 3, *; cough, #; acid reflux, $; cough-related acid reflux). Following re-initiation of PPI, the cough disappeared (cough level 1, cough reflex sensitivity 31.2 μM).

## Discussion and Conclusion

In case 2, although bronchial hyperresponsiveness to methacholine was noted, the patient was not thought to suffer from eosinophilic airway diseases such as bronchial asthma or CVA, since no eosinophils were found in bronchoalveolar lavage fluid, and neither bronchodilator therapy nor high dosages of glucocorticosteroid therapy improved the patient's cough. Although bronchial hyperresponsiveness is one of the characteristics of bronchial asthma, many clinically healthy volunteers exhibit high degrees of bronchial responsiveness. We considered his bronchial hyperresponsiveness unassociated with his cough.

The endoscopic findings and good response to PPIs initially suggested that these patients suffered from GERD-associated cough. However, 24-h esophageal pH monitoring revealed that most of the coughing was not temporally related to acid reflux episode. Although gastric acid is a most important factor in it, the development of esophageal damage depends on many factors including pepsin, bile acids, and pancreatic enzymes [5]. Since PPIs relieved their cough, if cough were the result of factors other than gastric acid, PPIs presumably inhibited them. However, this seems unlikely pharmacologically.

Because 24-h esophageal pH monitoring is quite invasive, the diagnosis of GERD-associated cough has usually been made based merely on the clinical efficacy of treatment with PPI [6]. We also had diagnosed GERD-associated cough based on clinical efficacy of this type prior to treating these two patients. Fortunately, these two patients gave us the opportunity to investigate their cough further. The findings obtained suggested that PPI might be effective not only for cough temporally related to gastric acid reflux but also for cough temporally unrelated to it. Although it is possible that the efficacy of PPIs involves a placebo effect, it was difficult to conclude this given their clinical course, in which cough was markedly improved after initiation of PPI, returned nearly to pretreatment level after discontinuation of PPI, and improved again following re-initiation PPI.

Given the findings of esophagitis, we could consider their suffering from GERD, but could not do GERD the etiology of their cough since most of their coughing was not temporally related to periods of acid reflux. Although it is believed that cough in GERD patients can be relieved regardless of whether it is temporally related to episodes of reflux or not, cough and GERD are common conditions and the likelihood of their co-existence by chance is high [7], GERD-associated cough should thus be diagnosed when the cough occurs simultaneously or within a few minutes of acid reflux. Patients should not be diagnosed as having GERD-associated cough just because they had GERD and their cough improved after taking PPIs. We suggest that the relationship between cough and reflux episodes requires investigation. In our cases, we could not consider cough GERD-associated since it occurred a few hours or more after reflux episodes. If it had occurred simultaneously or within a few minutes after reflux episodes, we would have diagnosed it as due to GERD. We suggest that though they definitively had GERD, their cough was not directly related to GERD.

Recent studies have indicated that PPIs have effects well beyond acid suppression, and have revealed many types of inflammatory cytokines in the esophageal mucosa of GERD patients [8]. Hamaguchi et al. [9] showed that PPIs can protect against esophageal inflammation via anti-inflammatory effects including inhibition of cytokine production, adhesion molecules expression, and neutrophil activation. Oribe et al. [10] showed that PPI, but not histamine H2 blocker, could directly decrease antigen-induced cough reflex hypersensitivity. These findings suggest that PPIs may act as new anti-tussive agents in treating chronic cough.

While the mechanism of improvement of cough without acid reflux remains unclear in detail, our most important finding is that PPI-responsive cough is not simply identical to cough temporally related to gastric acid reflux. The type of PPI-responsive cough we are proposing includes not only the same as our presenting cases, but also true GERD-associated cough. Thus, 24-h esophageal pH monitoring should be performed to determine the precise temporal relationship between cough and acid reflux if GERD-associated cough is to be diagnosed.

## Abbreviations

AC, atopic cough; ACE-I, angiotensin converting enzyme inhibitor; CVA, cough variant asthma; GERD, gastroesophageal reflux disease; PPI, proton pump inhibitor; SBS, sinobronchial syndrome

## Competing interests

The author(s) declare that they have no competing interests.

## Authors' contributions

All authors have read and approved the final manuscript. KN had primary responsibility for drafting and submitting the manuscript. HA was involved in the patients' clinical assessment and treatment.
